# Endoscopically assisted transoral resection of a Bailey type IV second branchial cleft cyst: A case report

**DOI:** 10.1097/MD.0000000000024375

**Published:** 2021-01-22

**Authors:** Shan Gao, Qin Xu, Qinchuan Yi

**Affiliations:** a Department of Otolaryngology-Head and Neck Surgery; b Department of Oncology, Zigong Fourth People's Hospital, Zigong, PR China.

**Keywords:** Bailey type IV, case report, parapharyngeal space, second branchial cleft cyst, transoral resection by endoscopic assistance

## Abstract

**Rationale:**

The diagnosis of type IV branchial cleft cyst (BCC) according to the Bailey classification is very challenging due to lack of specific clinical manifestations in the early stage of the disease. Here, we present the transoral surgical route of endoscopic resection of second BCC in the parapharyngeal space (PPS) with good outcomes.

**Patient concerns:**

A 21-year-old man with a 1-year history of snoring complained about sore throat for 1 month and a fever that lasted for 3 days.

**Diagnoses:**

On admission, physical examination revealed a temperature of 39°C, pain when swallowing accompanied with a lump sensation in the throat, and inability to open mouth more than 3 cm. Blood testing revealed 19.29 × 10^9^ white blood cells (WBCs)/L and 14.94 × 10^9^ neutrophils/L. A cervical computed tomography (CT) examination revealed a mass with liquid density of 6.2 × 4.0 × 7.7 cm^3^ in the left parapharyngeal space (PPS) and pharyngeal cavity stenosis. Postoperative pathology showed the existence of lymphoepithelial cysts (left PPS), which was in accordance with the diagnosis of BCC.

**Interventions:**

The patient was administered 1.5 g ceftazidime every 12 hours, anti-inflammatory drugs, and incision drainage was performed subsequently. Then, endoscopy-assisted resection of the left PPS was performed via the transoral route. We used low-temperature plasma and an 8-Fr Foley catheter with a water capsule during the surgery.

**Outcomes:**

After resection of the mass, the patient's blood results returned to within the normal range and his symptoms improved. Five days postoperatively, the incision made in the palatine arch of the pharynx opened up by 1 cm, and eventually the wound and laceration healed. Normal oral eating was restored, and no complications were observed.

**Lessons:**

Magnetic resonance imaging (MRI), and color Doppler ultrasound can be useful to diagnose BCC in PPS, which rarely occurs in the clinical setting. Extended endoscopy provides a satisfactory surgical field for trans-oral resection allowing complete resection of the BCC without serious postoperative complications.

## 1 Introduction

Head and neck tumors of the parapharyngeal space (PPS) are sporadic and have an incidence of approximately 0.5%. Based on previous retrospective findings, including 1293 cases,^[1,2]^ most PPS tumors are of salivary origin (45%), followed by neurogenic tumors (40%). Second branchial cleft cysts (BCCs) are developmental cyst and are the rarest type of PPS tumors. Bailey et al classified BCCs into 4 types based on location:^[3]^ Type I is located in the anterior edge of the sternocleidomastoid muscle and the deep surface of the latissimus dorsi muscle; type II is the most common type of BCC and is located behind the submandibular gland on the superficial surface of the sternocleidomastoid muscle and the lateral side of carotid space; type III BCC is located between the carotid bifurcation and the external carotid artery in the lateral wall of pharynx; and type IV is located in the interval of the pharyngeal mucosal space. The diagnosis of type IV BCC according to the Bailey classification can be very challenging due to the lack of specific clinical manifestations in the early stage of the disease; thus, this type of tumor can be easily misdiagnosed. Furthermore, considering the anatomical position of the PPS, the important neurovascular structure is complex, the operation is difficult to perform, and the associated risk of operation is high. In particular, when that tumor is very close to the skull base, it is difficult to expose the upper end, and attempting to separate the tumor without direct visualization can easily lead to the rupture of the cyst, resulting in incomplete resection and serious complications caused by the neurovascular injury.

With the development of endoscopic procedures, there is a better possibility to remove some tumors located in PPS via an intraoral approach. In the present study, we reported a single case of a patient with a giant BCC (with a diameter of approximately 8 cm, Bailey classification type IV) in the PPS who underwent endoscopic resection. We utilized low-temperature plasma and an 8-Fr Foley catheter with a water capsule during the surgery, which consequently improved the postoperative outcomes.

## 2 Case presentation

### 2.1 Clinical data

A 21-year-old man with a 1-year history of snoring who had a sore throat for 1 month and fever for 3 days was admitted at our hospital on September 20, 2018. Physical examinations revealed a body temperature of 39°C, no difficulty in breathing, hoarseness, and pain when swallowing, accompanied with a lump like sensation in the throat. No facial numbness was observed. He could not open his mouth more than 3 cm, the congestion in the pharyngeal mucosa was reddish, and the bilateral tonsils were hyperemic. The mucous membrane of the left palatal arch was swollen, smooth and the swelling crossed the midline. The patient had soft, weak neck, with pressure pain on the mass. A clear boundary was observed around the left submandibular gland. In addition, the patient had a history of upper respiratory tract infection. Peripheral blood testing showed 19.29 × 10^9^ white blood cells (WBCs)/L and 14.94 × 10^9^ neutrophils/L. All other values were within the normal range.

**Figure 1:**
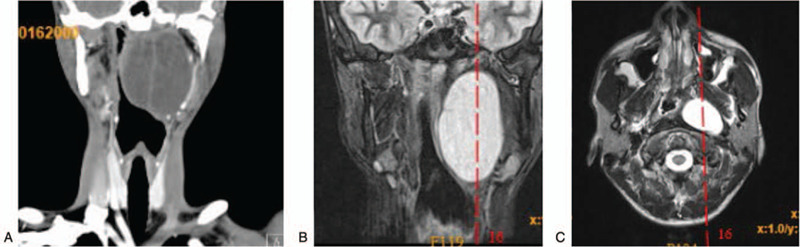
(A) Coronal computed tomography (CT) scan at first hospitalization showing a cystic lesion of 6.2 cm × 4.0 cm × 7.7 cm in the left parapharyngeal space, above the skull base, below the medial submandibular gland, protruding into the pharyngeal cavity, and resulting in pharyngeal cavity stenosis. (B–C) MRI (6 months later; second hospitalization) indicated that the dimensions of the left parapharyngeal space (left rear of the nasopharyngeal oropharynx plane) were approximately 5.1 cm × 2.9 cm × 7.6 cm with abnormal signal shadow, which is consistent with cystic lesions, protruding into the pharyngeal cavity, and resulting in pharyngeal cavity stenosis.

The patient was administered 1.5 g ceftazidime every 12 hours and anti-inflammatory drugs. Puncture incision and drainage was performed at the most swollen portion on the left side of his mouth and pharynx. His symptoms remarkably improved after 200 ml of the purulent discharge was drained. The patient then underwent cervical computed tomography (CT) examination, which revealed a liquid density of 6.2 × 4.0 × 7.7 cm^3^ in the left parapharyngeal space and pharyngeal cavity stenosis. The bilateral pharyngeal recess, pharyngeal orifice of the eustachian tube, skull base, and the cervical spine remained intact, and the cervical lymph nodes were not enlarged (Fig. 1A). Given the possibility of complication of this infection involving the posterior pharyngeal and left parapharyngeal space, resection of the mass was suggested after anti-infection treatment. After 5 days of open drainage and treatment for infection, the patient's symptoms were mostly alleviated. Results of the peripheral blood examination at this stage showed that the patient's leukocyte (5.7 × 10^9^/L) and neutrophil (3.58 × 10^9^/L) counts returned to the normal range, and bacteriological culture of the puncture fluid yielded no microbial growth.

On March 13, 2019, the patient complained of having a pharyngeal foreign body sensation, yet he had no fever, swallowing pain, expiratory dyspnea, and no obstruction when opening his mouth. He was admitted to the hospital again and underwent CT and magnetic resonance imaging (MRI). The cervical spine CT scan showed a well-defined, homogeneous, cystic mass in the left PPS with the dimensions 5.2 cm × 4.2 cm × 7.6 cm; this mass displaced the carotid sheath and its structures on the posterolateral side. An MRI of the pharynx and larynx revealed that the volume of the left PPS was approximately 5.1 cm × 2.9 cm × 7.6 cm, with an abnormal signal shadow, uneven signal intensity, high signal intensity on T2WI, high signal intensity with lipid suppression, clear boundary, separation of internal shadow, and limited diffusion. The left pharyngeal recess could not be seen, whereas the right pharyngeal recess, the pharyngeal orifice of the eustachian tube, and the skull base and cervical vertebrae were not damaged (Fig. 1B and C). Results of the blood testing showed 5.00 × 10^9^ leukocytes/L and 2.73 × 10^9^ neutrophils/L.

### 2.2 Surgical procedure and intraoperative findings

The treatment procedure and possible complications (exposure difficulties or potential massive intraoperative bleeding) were explained in detail to the patient, after which he signed a written informed consent. Endoscopy-assisted resection of the left PPS via the transoral route was performed on March 15, 2019, after the patient was placed under general anesthesia. The patient received 1.5 g of ceftazidime 30 minutes before the start of the operation. Briefly, his head was pushed back and an opening device manufactured by Davis Stage Cementing Collars and Equipment (Houston, TX, USA) was used. The device was placed in the same position as that used for adenoid removal, following which the catheter was inserted into the nasopharynx via the contralateral nasal cavity and fixed in place by hanging on to the soft palate. Consequently, the mucosal and submucosal tissues were cut in the arc line (between the most protruding soft palate on the left side and the posterior level of the second molar on the left side of the jaw) with the iso-ionizer MC403 (Chengdu Meichuang Co., Ltd., Chengdu, China). A 0° nasal endoscope (18 cm in length, 4 mm in diameter; Karl Storz SE & Co. KG, Tuttlingen, Germany) was used. The palatine and lingual muscles were then bluntly separated, as well as the supra-pharyngeal constrictor muscle located in the medial PPS, and the cyst wall and with the outer capsule of the tumor capsule by electrocoagulation. To prevent the rupture of the thin capsule wall and appearance of the residual recurrence of the capsule wall during separation, middle curved hemostatic forceps were used to properly separate the space around the capsule wall. Consequently, an 8-Fr double cavity water capsule enclosed in a Foley catheter was inserted along the lateral dorsal membrane of the cyst. Next, 3 to 5 ml of water was injected into the capsule to enlarge the space, allowing the hemostatic forceps to adequately re-separate the surrounding space after the bipolar electrocoagulation from the surrounding tissue under the guidance of direct vision. Water was then pumped through the catheter to enlarge the capsule, and the hemostatic forceps and the water capsule were alternately moved forward to expand the cavity. This created a satisfactory visual field to observe, distinguish, and protect the important blood vessels and nerves, and achieve meticulous blood-free separation for endoscopic endonasal surgery.

The medial, anterior, and superior skull base, and the lateral and posterior regions of the tumor were separated. Bipolar electrocoagulation or gauze compression were used to stop the bleeding in case of intraoperative bleeding, ensuring smooth proceedings. Nevertheless, during the separation of the lower pole of the cyst, the cyst wall was accidentally ruptured when the root pedicle was severed by a plasma knife. After aspirating approximately 200 ml of milky white capsular fluid, the capsule was completely removed along the capsule wall guided by endoscopic vision, and the cavity was carefully examined. After sufficient hemostasis was achieved, the cavity was repeatedly washed with 2% hydrogen peroxide solution, dilute iodophor, and saline. Finally, the left tonsil was resected. No active bleeding was found in the cavity, and the cavity was sprayed with ceftazidime powder spray. One piece of yarn and 1 piece of gelatin sponge were used for filling the cavity, and the PPS was closed and the pharyngeal contractile muscle, submucous membrane, and mucous membrane were individually sutured. One rubber drainage tube was retained in the subpolar incision of the surgical cavity, and the other end of the thread from the left nasal cavity was fixed in the nasolabial groove to prevent shedding. Intraoperative bleeding was approximately 20 ml. To prevent postoperative dyspnea caused by swelling of the oral and pharyngeal mucosa, a prophylactic tracheotomy was performed and antibiotics and steroid hormones were administered.

## 3 Outcomes

**Figure 2:**
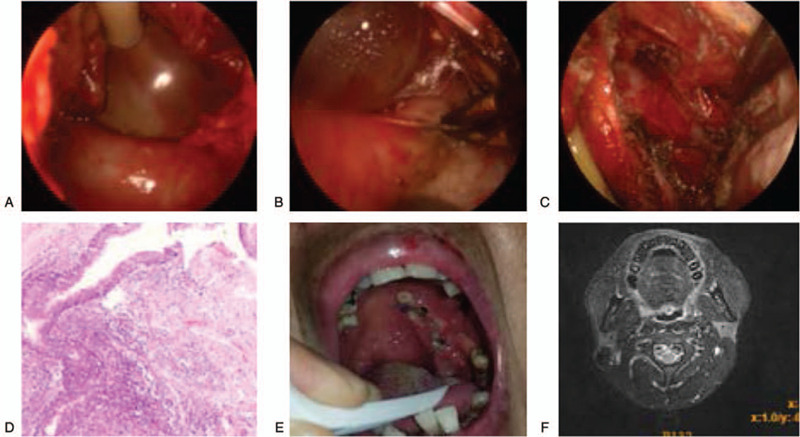
(A) An 8-Fr Foley catheter was inserted along the lateral dorsal membrane of the cyst, and 3- to 5-ml of water was injected into the capsule to enlarge the space. (B) Separation of peripheral blood vessels and nerves along with the enlarged space. (C) Complete removal of the pharyngeal space cyst, and the surrounding blood vessels and nerves were preserved in the cavity. (D) Postoperative pathology showed a lymphoepithelial cyst in the left pharyngeal space (HE ×200). (E) Pharyngeal examination performed half a month after discharge revealed that the incision of the palatal arch was closed and it had completely healed. (F) MRI scan performed 6 months after the operation.

The limitation of mouth opening occurred immediately after the surgery due to stimulation of internal pterygoid muscle, but this gradually resolved after a few days. The drainage strip and normal flow nasal cannula were withdrawn at 3 and 5 days postoperatively, respectively. Five days postoperatively, the incision made in the palatine arch of the pharynx opened up by 1 cm. Subsequently the wound and laceration healed, normal oral eating was restored, and no complications were observed. After 1 week, the patient recovered and was discharged from the hospital. Postoperative pathology showed the presence of lymphoepithelial cysts (left PPS), which was in accordance with the diagnosis of BCC (Fig. 2). A follow-up examination conducted 15 days after discharge revealed that the palatal region that was closed by making an incision had fully healed, and the patient did not experience disrupted sleep due to snoring anymore. At 6 months postoperatively, MRI showed no recurrence (Fig. 2). At the time of writing this paper, the patient is still being followed up and no special discomfort has been noted since the operation.

## 4 Discussion

The main findings of this study are as follows: although cervical incision surgery is the most common approach to treat BCC in PPS, for this particular case, endoscopy-assisted minimally invasive surgery with a catheter water capsule was found to be the better treatment option. Compared to conventional cervical surgery that entails creating a large incision, the endoscopy-assisted and catheter-based surgical approach can fully expose the surgical field, reduce surgical injury, and complete resection without inducing scar formation. A CT, MRI, and color Doppler ultrasound can be useful to diagnose a BCC in PPS, which is a rare occurrence in the clinical setting.

Most PPS tumors are benign and asymptomatic, while a small percentage of these tumors may be malignant. Due to their anatomic location, PPS tumors represent a therapeutic challenge and can be diagnosed using physical and imaging examinations.^[4]^ Most of the PPS tumors are of salivary gland origin. Some researchers have suggested that shifting of the soft palate to the opposite side and the bulging of the pharyngeal wall as well as swallowing obstruction and in some cases dyspnea, tinnitus, ear tightness, and hoarseness may be indicators of PPS tumor.^[5,6]^ Neurogenic tumors are common when the tumor is located in the PPS. These tumors often manifest in the deeper part of the ipsilateral submandibular gland and as medial mass of the lower jaw. When the tumor compresses the adjacent intracranial nerve and sympathetic trunk, then hoarseness, dysphagia or dyspnea, tinnitus and ear tightness, Horner sign, and soft palatal deviation caused by ipsilateral pharyngeal lateral wall compression can appear.^[7]^ Further growth of the tumor can lead to invasion into the brain through the foramen ovale, anterior hypoglossal nerve tube, jugular foramen, and other structures.^[8–10]^

The cause of BCC remains unclear. Some researchers believe that BCC originates from the residue of branchial cleft epithelial cells; others consider it to be caused by 1 or more factors such as incomplete branchial sulcus closure, rupture of the closure membrane between the branchial sulcus and pharyngeal capsule, abnormal development of the branchial organ, and retention of the cervical sinus. Paczona et al^[11]^ explained the existence of parapharyngeal cysts without sinus or fistula. They found that the branchial arch appears at the beginning of the fourth week in human embryos, and is bilaterally symmetrical. One to 4 pairs of branchial arches appear at the end of week 4, while the fifth pair is rarely observed in humans. There are 5 pairs of branchial sulci between the arches. The first pair of branchial arches develops into the maxillary eminence and mandibular eminence, which are involved in the formation of facial surfaces. The second pair is involved in the formation of the hyoid bone and neck, and it grows rapidly to the tail, gradually covering pairs 3 and 4. The space between the second pair and the other lower branchial arch spaces is called the cervical sinus, and this undergoes atresia as part of normal development and forms the neck. If abnormalities in the normal embryonic development lead to non-closure of the cervical sinus, the residual epithelial tissue forms a branchial cyst. Depending on the sites, if the cyst is located between the mandibular angle and the hyoid, it originates from the second branchial cleft, and this type of BCC has a high incidence of occurrence. Bailey divided the second BCC into 4 types according to its relationship with cervical tissue. Of the 4 types in the Bailey classification, type IV is the rarest. Some researchers consider that the retained branchial plate can cause ectoderm- endoderm adhesion in the absence of mesoderm arch insertion or branchial plate mouth rupture, and that the epithelial lining of the cyst can be formed under the endothelium of the palatine tonsil, and no fistula can enter the oropharynx.^[11]^ Some researchers have proposed the theory of “benign lymphoepithelial cyst,” considering that most cyst specimens have lymphoid tissue.^[12]^ It has been considered that epithelial cells of the salivary gland that became trapped in the cervical lymph nodes during the embryonic stage have cystic lymph node lesions, or “benign lymphoepithelial cysts.” Furthermore, Jehad et al reported the rare case of a second BCC in a 9-year-old child and found lymphoid accumulation in the cystic wall with scaly epithelial lining.^[13]^

The second BCC grows slowly and often discovered incidentally as painless masses in the neck or parotid gland, which can be accompanied by intermittent distension and pain in the neck or pharynx, especially when swallowing. When complicated with upper respiratory tract infection and inflammation, the swelling becomes aggravated with obvious pain, the mass increases rapidly, and can even rupture. Some patients develop fever and hoarse voice or dyspnea. A combination of cervical CT and MRI scans can be used to examine the approximate location, size, range, smoothness and texture of the tumors, and the anatomical relationship of the tumors with the surrounding blood vessels and nerves. When infection or bleeding occurs, the fibrosis of the capsule wall thickens, and a large number of cholesterol crystals and foreign body giant cell reactions occur in the wall. Consequently, the capsule wall becomes more closely adhered to the surrounding tissue, leading to difficulty in the separation of the capsule wall during surgery.

In the present study, we report a patient with a giant BCC (approximately 8 cm in diameter) in the PPS, who was treated with endoscopic resection. After conservative treatment, cervical spine CT showed a well-defined, homogeneous, cystic mass in the left PPS with the dimensions 5.2 cm × 4.2 cm × 7.6 cm, displacing the carotid sheath and its structures on the posterolateral side. The cystic mass compressed the left tonsil anteriorly, leading to severe narrowing of the oropharyngeal passage. Although the patient's symptoms were obviously alleviated, one-stage resection was recommended for complete resection and to prevent recurrence. Hence, endoscopy-assisted oral resection of the cyst was selected based on the following considerations:

1. The cysts were big and located on the medial side of the internal carotid artery, resulting in the outward displacement of large vessels and intracranial nerves. Under endoscopic guidance, the main vessels, nerves, small lesions, and microvessels were carefully identified. This would not be possible using the conventional cervical approach, which may significantly increase the risk of massive bleeding and adjacent craniocerebral nerve (IX–XII) injury.^[14,15]^
2. Low-temperature plasma equipment can be used to wash, stop hemostasis, cut synchronously, and solidify the small blood vessels around the tumor, which can effectively reduce bleeding and shorten the operation time. The tumor can be exposed by a submucous incision of pharyngeal contractile muscle on the side of the pharyngeal wall. The submucous incision requires less suture time. Although the cervical approach has a wide field of vision, electrocoagulation and suction devices should be used during the operation, and the skin, subcutaneous tissue, and latissimus cervicalis muscle should be cut open in turn and then sutured after the operation. It is obvious that the endoscopic transoral approach has great advantages over the cervical approach in terms of operation time and bleeding volume.
3. Simple incision, puncture, and cyst aspiration or injection of sclerosing agents can lead to the residual cyst epithelium retention and increase the risk of recurrence. A previous study reported the resection of a large tumor after volume reduction by opening the capsule to extract part of the capsular fluid. Similarly, there have been successful reports of ethanol injection therapy in patients who do not wish to undergo operation.^[4]^ In these patients, the BCC was located in the narrow PPS and the tension was very high. Once the capsule was opened, all the fluid was instantly ejected, leading to the unclear identification of the capsule wall epithelium and recurrence of the residual cyst wall epithelium. In order to achieve complete resection, we used hemostatic forceps to “explore the route,” and Foley catheter rotation and water capsule separation for cavity expansion were used for “soft” separation and pole electrocoagulation was used for separation of adhered tissues. The alternative “move-forward” method can be used to achieve complete resection of cysts along the capsule. Although the capsule fluid was ejected by accidental rupture of the capsule by ultrasonic knife when the polar root pedicle was severed, it ensured complete removal of the residual cyst epithelium of the root pedicle under endoscopic guidance.
4. The oral approach provides the best aesthetic effect, as it avoids the scar and functional impairment caused by a long neck incision. Yet, when using this approach, the styloid and surrounding adjacent muscles should be used as anatomical landmarks during medial approach surgery. Recent studies have described the treatment of cyst bagging via oral approach,^[2]^ but the author believes that recurrence may still occur postoperatively due to infection and pouch mouth atresia. In this case, although the operative cavity was filled with absorbable hemostatic material and indwelling drainage strip after cystectomy, the incision still opened up after operation. To sum up, we believe the negative pressure drainage tube should be retained to promote the healing of the PPS and to prevent incision opening after the surgery. The upgrade of incision grade makes it necessary to combine broad-spectrum antibiotics with antianaerobic antibiotics after caliber surgery, and steroid hormones are used to prevent dyspnea due to edema.^[16]^ Meanwhile, based on our personal experience, we find that general anesthesia via contralateral nasal intubation is more beneficial than direct oral intubation for this surgery. Broad-spectrum antibiotics with antianaerobic antibiotics, and steroid hormones can be used to prevent dyspnea due to edema.^[16]^

In summary, a CT, MRI, and color Doppler ultrasound are useful techniques to diagnose BCC in PPS, which is rare in the clinical setting. The extended use of endoscopy and 8-Fr catheter water capsules for separation of the outer wall of the cyst are satisfactory surgical approaches for trans-oral resection, allowing for complete resection of the BCC with no serious postoperative complications. However, preventive tracheotomy is still recommended to prevent the occurrence of postoperative complications of dyspnea caused by bleeding and swollen mucous membrane.

## Author contributions

**Conceptualization:** Shan Gao, Qin Xu.

**Data curation:** Shan Gao, Qinchuan Yi.

**Formal analysis:** Shan Gao, Qin Xu, Qinchuan Yi.

**Investigation:** Shan Gao.

**Project administration:** Qin Xu.

**Writing – original draft:** Shan Gao.

**Writing – review & editing:** Qin Xu, Qinchuan Yi.

## References

[1] Kuet ML Kasbekar AV Masterson L Management of tumors arising from the parapharyngeal space: a systematic review of 1,293 cases reported over 25 years. Laryngoscope 2015;125:1372–81. 25448637 10.1002/lary.25077

[2] Riffat F Dwivedi RC Palme C A systematic review of 1143 parapharyngeal space tumors reported over 20 years. Oral Oncol 2014;50:421–30. 24589290 10.1016/j.oraloncology.2014.02.007

[3] Bailey H Branchial Cysts and Other Essays on Surgical Subjects in Faciocervical Region. 1929;London: Lewis, 56.

[4] Basaran B Polat B Unsaler S Parapharyngeal space tumours: the efficiency of a transcervical approach without mandibulotomy through review of 44 cases. Acta Otorhinolaryngol Ital 2014;34:310–6. 25709146 PMC4299156

[5] Garcia-Ortega DY Gomez-Pedraza A Luna-Ortiz K Parapharyngeal space lipomatosis with secondary dyspnea, disphagia and disphonia. Int J Surg Case Rep 2015;15:54–6. 26318127 10.1016/j.ijscr.2015.08.016 PMC4601961

[6] Yousem D Head & Neck Imaging: Case Review Series. 4th ed. 2015;Philadelphia: W.B.Saunders, 354.

[7] Bist SS Luthra M Agrawal V Giant parapharyngeal space pleomorphic adenoma causing acute airway obstruction. Oman Med J 2017;32:240–2. 28584606 10.5001/omj.2017.44 PMC5447789

[8] Laturiya R Kasim JS Jankar AS Pleomorphic adenoma of minor salivary gland arising de novo in the parapharyngeal space- a rare case report. J Clin Diagn Res 2016;10:ZD01–3. 10.7860/JCDR/2016/18435.7356 PMC4843395 27135010

[9] Lee JE Hong HS Chang KH Solitary fibrous tumor of the post-styloid parapharyngeal space. Acta Radiol Short Rep 2014;3:2047981614536158. 25298872 10.1177/2047981614536158 PMC4184415

[10] Metgudmath RB Metgudmath AR Malur PR Surgical management of parapharyngeal space tumors: our experience. Indian J Otolaryngol Head Neck Surg 2013;65:64–8. 24427618 10.1007/s12070-012-0508-7 PMC3718926

[11] Paczona R Jori J Czigner J Pharyngeal localizations of branchial cysts. Eur Arch Otorhinolaryngol 1998;255:379–81. 9783138 10.1007/s004050050082

[12] Wild G Mischke D Lobeck H The lateral cyst of the neck: congenital or acquired? Acta Otolaryngol 1987;103:546–50. 2441567

[13] Al Sukhun J El Naggar M Unusual presentation of a large multilocular second branchial cleft cyst. J Craniofac Surg 2019;30:1772–3. 31033768 10.1097/SCS.0000000000005506

[14] Vidhyadharan S Krishnan S King G Transoral robotic surgery for removal of a second branchial arch cyst: a case report. J Robot Surg 2012;6:349–53. 27628477 10.1007/s11701-011-0331-2

[15] Goldenberg D Ondik MP DaVinci robot-assisted transcervical excision of a parapharyngeal space tumor. J Robot Surg 2010;4:197–9. 27638760 10.1007/s11701-010-0201-3

[16] Pilolli F Giordano L Galli A Parapharyngeal space tumours: video-assisted minimally invasive transcervical approach. Acta Otorhinolaryngol Ital 2016;36:259–64. 27734977 10.14639/0392-100X-709 PMC5066460

